# Being Uncertain in Chromatographic Calibration—Some Unobvious Details in Experimental Design

**DOI:** 10.3390/molecules26227035

**Published:** 2021-11-21

**Authors:** Łukasz Komsta, Katarzyna Wicha-Komsta, Tomasz Kocki

**Affiliations:** 1 Chair and Department of Medicinal Chemistry, Faculty of Pharmacy, Medical University of Lublin, Jaczewskiego 4, 20-090 Lublin, Poland; 2 Chair and Department of Clinical and Experimental Pharmacology, Faculty of Medicine, Medical University of Lublin, Jaczewskiego 8b, 20-090 Lublin, Poland; katarzyna.komsta@umlub.pl (K.W.-K.); tomasz.kocki@umlub.pl (T.K.)

**Keywords:** calibration, optimality, regression, chemometrics, experimental design, uncertainty

## Abstract

This is an introductory tutorial and review about the uncertainty problem in chromatographic calibration. It emphasizes some unobvious, but important details influencing errors in the calibration curve estimation, uncertainty in prediction, as well as the connections and dependences between them, all from various perspectives of uncertainty measurement. Nonuniform D-optimal designs coming from Fedorov theorem are computed and presented. As an example, all possible designs of 24 calibration samples (3–8, 4–6, 6–4, 8–3 and 12–2, both uniform and D-optimal) are compared in context of many optimality criteria. It can be concluded that there are only two independent (orthogonal, but slightly complex) trends in optimality of these designs. The conclusions are important, as the uniform designs with many concentrations are not the best choices, contrary to some intuitive perception. Nonuniform designs are visibly better alternative in most calibration cases.

## 1. Introduction

Chromatography is widely used for the quantitative analysis of a diverse array of samples, often inside complex matrices. Validation of a quantitative method requires a proper calibration step [1,2,3], defined as the estimation of dependence between the analyte amount and a method response (peak height or area) [4,5,6]. As most of routine analysis is still based on spectrophotometric detection with absorbance measurement, the calibration curve is in most cases linear. Nevertheless, a small nonlinearity is often present, forcing analysts to fit a quadratic equation.

The calibration equation is obtained from a set of calibration samples with a (most often least squares) regression method. The obtained equation should be understood as an estimator, which approximates true (but never known) value. Estimation is always connected with an uncertainty [3], which depends on various factors. One of these factors is the design of calibration experiments.

There are numerous papers about calibration theory in context of linear regression considerations and assumptions, a strong effort was also put in the literature on dealing with situations when there is a failure to meet some of them. Two papers of Baumann and Watzig [7,8], as well as a general tutorial by Lee and McAllister [9] could be recommended as a good starting point.

On the contrary, there is a lack of papers dealing with optimality of design during the calibration process, written for chemists in their perspective. Papers touching upon this area have been mainly published in statistical journals and written for statisticians (including dozens of impractical math formulas) without clear conclusions for analysts (they are cited in this paper in the appropriate places). Scheffe [10] is a good first choice for the interested reader.

In this paper we would like to touch upon these problems, emphasizing several not so obvious details in the calibration process, in the most accessible way we can. We hope that this article will expand knowledge about the calibration process and will result in more proper design of future chromatographic methods.

## 2. Theory

### 2.1. Classical Formulation of Simple Linear Regression

We will introduce only the most important formulas, essential to understand everything given further in context of the design [7,8,11]. Let us start with a classic formula of multiple linear regression, where one response variable is modeled as a linear combination of several variables:

$$y_{i}=\beta_{0}x_{i0}+\beta_{1}x_{i1}+\beta_{2}x_{i2}+\cdots +\beta_{p}x_{ip}+\varepsilon_{i}$$

where $\varepsilon_{i}$ can be interpreted as some random error with normal distribution. The linear or polynomial calibration can be perceived as a special case of such problem:

$$y_{i}=\beta_{0}+\beta_{1}x_{i}+\beta_{2}x_{i}^{2}+\cdots +\varepsilon_{i}$$

In this special case, $x_{i0}$ always equals 1 (the intercept term) and the subsequent polynomial coefficients contain the same x, but raised to a power. The chromatographic response is then modeled as a linear combination of one (linear) or several (polynomial, i.e., quadratic, cubic etc.) powers of the same concentrations. Natural processes are rarely polynomial, but polynomial fitting can model almost all nonlinear functions in a flexible way (by finding its Taylor expansion).

It should be mentioned that there is a possibility to remove the intercept term, forcing the calibration curve to go through origin. Although this idea could be perceived as advantageous (as the response should be zero without the analyte), calibration should always include the intercept. Its value and significance is an important diagnostic tool [12]. In general, in most of methods the intercept term should be insignificant, and its significance indicates a problem with calibration. Nevertheless, there are some analytical methods (for instance TLC with densitometry), where offset of the response is possible, and significance of the intercept term does not mean that the method is elaborated in a bad way.

Although the polynomial equation is not represented as a straight line, the fitting is done as a linear regression problem. In practice, it is very advantageous. One can analytically find the exact solution, opposite to nonlinear regression, when starting values must be explicitly given and the whole process is only a numerical iterative optimization. In most chromatographic cases, a quadratic equation can deal enough well with nonlinearity, cubic equation is used extremely rarely. Therefore, we will describe only linear and quadratic cases in this paper.

The regression equation can be rewritten in vector form $y_{i}=x_{i}^{T}\beta +\varepsilon_{i}$, where β is the vector of coefficients of the fitted equation. Going one step further, we reach matrix representation of the calibration problem:

y=Xβ+ε

Here y is a vector of noticed responses, whereas X is a matrix containing ones in the first column (intercept), concentrations in the second one and optionally squares of concentrations in the third one (and so on when higher order polynomial is fitted). We need to solve this equation for a vector β, which minimizes the error ε in a least squares sense: sum of squared errors (residuals) should be as smallest as possible.

Not deeply diving in the mathematical theory, there is an exact solution. Multiplying both sides by $X^{T}$ (transpose of X) one can obtain $\left(X^{T}X\right)\beta =X^{T}y$. Dividing then both sides by the crossproduct $X^{T}X$ (which is equivalent to multiplying both sides by its inverse ${\left(X^{T}X\right)}^{-1}$), one can obtain the well-known formula:

$$\beta ={\left(X^{T}X\right)}^{-1}X^{T}y$$

As this is a matrix formula, it is correct for any number of calibration points (as well as any order of fitted polynomial). There is also a possibility to remove intercept term from the equation easily, by deleting the first column of X (containing ones).

The obtained β is an estimator of the relationship in the infinite population. By multiplying this estimator by the original concentration matrix **X**, we can obtain values computed from the obtained equation, which are called “fitted” values:

$$\hat{y}=X\beta =X{\left(X^{T}X\right)}^{-1}X^{T}y$$

This is equivalent to the multiplication of y by some matrix $H=X{\left(X^{T}X\right)}^{-1}X^{T}$, called hat matrix. This process can be understood as a projection to some limited subspace spanned by the fitted model.

The differences between original and fitted values ($\hat{y}-y$) are called the residuals, and the regression method finds the subspace of the model which minimizes sum of their squares. From a geometrical point of view, this is equal to minimization of information inside the orthogonal complement of the fitted model. The information in residuals is “lost”, in sense that it does not contribute to the model.

The fitted calibration line has a cognitive value only under precisely defined circumstances [12], and one of main requirements is that the residuals contain only random, homoscedastic and normally distributed error (a noise, without any information). To satisfy this condition, several issues must be assumed:

1. The modeled equation must match the real calibration dependence. This assumption is not valid when a straight line is fitted to a curvilinear dataset (instead of quadratic or cubic equation). Then, the residuals contain the whole nonlinearity pattern instead of a random error. It causes the introduction of a strong systematic error to the predicted values: the fitted equation does not estimate anything serious. The most frequently used validation criteria: Pearson’s correlation coefficient *r*, as well as coefficient of determination *R*^2^ are only measures of the error’s magnitude [13]: the randomness of the error is neglected by them. Therefore, when their value is close to 1, it does not indicate that the model is sufficient to describe the calibration dependence and that all assumptions are fulfilled [14].
2. There is no error in *x*: concentrations are known without any uncertainty. This cannot be achieved in practice, so calibration samples should be prepared as carefully, as possible. There are some approaches that include this error into the model [15,16]. It substantially makes the mathematical background much more complex, so they did not reach much attention in practice.
3. The error is homoscedastic (the variance of the error does not depend on the concentration of analyzed compound). This can be checked with visual inspection of residuals plot, as well as by Bartlett test on residual groups. In case of heteroscedascity, appropriate weights should be used in regression [17,18]. The most reasonable weighting is the reciprocal of the concentration, as the error is proportional to it. This is frequently a case in chromatography, when the injection volume remains the same, but calibration is done with increasing concentrations. When standard addition is performed, the error distribution change—this topic lies beyond the area of the current paper, and interested readers can be referred to [19,20]. Another important factor is also the use of certified reference materials [21].
4. The distribution of residuals should be as similar as possible to that of normal distribution. The attention should be put especially when the results were transformed. Although the logarithmic transformation seems to be good solution to deal with nonlinearity, it also transforms the errors [22,23]. Therefore, in most cases, quadratic regression is better than the combination of the linear regression and the transformation.

### 2.2. Uncertainty of the Regression Estimates

The variance of the residuals

$$\sigma^{2}=\frac{\sum_{i=1}^{n}{\left({\hat{y}}_{i}-y_{i}\right)}^{2}}{n-2}$$

(note n−2 in the denominator, as this is the real degree of freedom here) is the basic and most important measure of the response error. If the aforementioned assumptions are fulfilled, it contains only the noise, so it can be interpreted as an uncertainty of approximation of the real dependence, in infinite population from a finite calibration set.

The coefficients of the fitted equation are estimated with some own and individual uncertainties, which differ from $\sigma^{2}$. These errors come from multiplying $\sigma^{2}$ by the elements of the inverse of the covariance matrix:

$$\Sigma =\sigma^{2}{\left(X^{T}X\right)}^{-1}=\left[\begin{array}{cc} \sigma_{0}^{2} & \sigma_{01}\\ \sigma_{01} & \sigma_{1}^{2} \end{array}\right]$$

The above form of the matrix is for linear model; it is then 2 × 2 matrix with variances of $\beta_{0}$ and $\beta_{1}$ on its diagonal, and the covariance between them duplicated in the other cells (which tells us that the matrix is symmetric. For quadratic regression it will be quite larger:

$$\Sigma =\sigma^{2}{\left(X^{T}X\right)}^{-1}=\left[\begin{array}{ccc} \sigma_{0}^{2} & \sigma_{01} & \sigma_{02}\\ \sigma_{01} & \sigma_{1}^{2} & \sigma_{12}\\ \sigma_{02} & \sigma_{12} & \sigma_{2}^{2} \end{array}\right]$$

These formulas bring us an important conclusion. The error of the estimation is dependent on an error in the response (which cannot be changed as we have no influence on it), but also depends on the chosen concentrations, which are placed in the X matrix. The concentrations can be changed by the analyst, so it is possible to design the calibration in the way to minimize this error as much as possible. The second conclusion is: the estimators are intercorrelated. It can be a surprise, as this covariance is rarely given by statistical software in the regression output.

For the linear regression, the elements of ${\left(X^{T}X\right)}^{-1}$ can be quite simply computed:

$${\left(X^{T}X\right)}^{-1}=\left[\begin{array}{cc} \sum x^{2} & -\sum x_{i}\\ -\sum x & n \end{array}\right]\cdot \left(\frac{1}{S_{xx}}\right)$$

where $S_{xx}=\sum {\left(x-\bar{x}\right)}^{2}$, and $\bar{x}$ is the arithmetic mean of concentrations. So, it can be seen that the error lowers when variance of concentrations (in calibration design) increases. The slope estimation error is independent on the absolute value of concentrations, whereas the intercept error and the covariance are proportional to these values and increase together with their value.

The most important detail to understand now, is that uncertainties of the coefficients depend on various factors-there is no way to optimize them all at the same time. Let us summarize the best solutions for each uncertainty separately (without diving into mathematical background of the optimization behind):

1. To minimize the slope uncertainty, one should take concentrations with as large variance, as possible. The ideal solution would be to make the first half measurements at zero and the second half at the highest concentration [24].
2. To minimize the intercept uncertainty, one should measure one observation at the highest concentration and the other measurements should be done at concentration equal to zero. More reasonable solutions are available only when it is allowed to use negative concentrations, which does not happen in practice.
3. It is possible to get rid of the covariance between uncertainties—to achieve this goal, the concentrations should be symmetrical around zero (with mean equal to zero). Without negative concentrations, the lowest covariance is achieved in the same case as in (2).

Now let us focus on another details. The uncertainty does not depend on the coefficients of the obtained equation. It could be perceived as an obvious fact, but the linear regression is an exception. When fitting any nonlinear function to the data points, one always ends up with uncertainty formulas containing the fitted coefficients [25,26,27,28]. This leads to an interesting paradox—one should know the solution before designing the experiment (allowing the solution to be known). This is the one of many reasons why we prefer the linear regression in calibration.

### 2.3. Design Optimality

Instead of thinking which uncertainty is most important to optimize, one could optimize some overall variance criterion. Various criteria were proposed, and in the case of calibration the following approaches can be used [29,30]:

1. D-optimality, which minimizes the determinant $\left|{\left(X^{T}X\right)}^{-1}\right|=1/S_{xx}$, (which is equal to maximizing determinant of $X^{T}X$). Optimizing experiment in this way forces us to use the same strange design as when minimizing slope uncertainty.
2. C-optimality, minimizing the uncertainty of some linear combination of fitted coefficients, for example the uncertainty of the root of fitted line (such a parameter is often computed in lipophilicity measurements). In linear regression, the mean of *x* values must be equal to the place of the root. Not so useful idea-we are led again to the paradox, as we must know the answer before designing the experiment.
3. A-optimality, minimizing the trace (sum of diagonal values) of ${\left(X^{T}X\right)}^{-1}$. It can be seen, that this idea ends with the same solution, as minimizing the uncertainty of the intercept.
4. T-optimality (maximizing the trace of $X^{T}X$), as well as E-optimality (maximizing the minimum eigenvalue of $X^{T}X$), leading to measurement of all but one points at maximum concentration and the remaining point at zero (reverse idea than the intercept case).

The focused reader could ask now, why optimizing any uncertainty ends up with such a strange design, which is unused in calibration practice. It is obvious that, the calibration cannot be done at a concentration equal to zero: although the analysis of a blank response is the common practice in method validation, the results do not have any reliable information for modeling the calibration dependence. So, in the practice, the range of the calibration should be transformed, placing the left boundary at some small concentration instead of blank response.

Even if we did so, it could still look very strange to measure two concentrations only: a small one and the largest one (even with many repetitions). The intuitive approach is to measure something in the middle. The intuition gives us a good answer here, as two-point design assumes explicitly the linearity. Two concentrations simply do not allow any nonlinearity evaluation and cannot be fitted to quadratic equation if necessary.

### 2.4. Uncertainty in Quadratic Regression

So let us perform analogous journey through quadratic model. The information matrix for this case looks like in the following way:

$$X^{T}X=\left[\begin{array}{ccc} n & \sum x & \sum x^{2}\\ \sum x & \sum x^{2} & \sum x^{3}\\ \sum x^{2} & \sum x^{3} & \sum x^{4} \end{array}\right]$$

and the further computations become much more complicated:

$$\left|X^{T}X\right|=n(\Sigma x^{2}\Sigma x^{4}-\left(\Sigma x^{3}{)}^{2}\right)-\Sigma x\left(\Sigma x\Sigma x^{4}-\Sigma x^{2}\Sigma x^{3}\right)+\Sigma x^{2}(\Sigma x\Sigma x^{3}-\left(\Sigma x^{2}{)}^{2}\right)$$

$${\left(X^{T}X\right)}^{-1}=\left[\begin{array}{c} \Sigma x^{2}\Sigma x^{4}-{\left(\Sigma x^{3}\right)}^{2}\;\Sigma x^{2}\Sigma x^{3}-\Sigma x\Sigma x^{4}\;\Sigma x\Sigma x^{3}-{\left(\Sigma x^{2}\right)}^{2}\\ \Sigma x^{2}\Sigma x^{3}-\Sigma x\Sigma x^{4}\;n\Sigma x^{4}-{\left(\Sigma x^{2}\right)}^{2}\;\Sigma x\Sigma x^{2}-n\Sigma x^{3}\\ \Sigma x\Sigma x^{3}-{\left(\Sigma x^{2}\right)}^{2}\;\Sigma x\Sigma x^{2}-n\Sigma x^{3}\;n\Sigma x^{2}-{\left(\Sigma x\right)}^{2} \end{array}\right]\cdot \left(\frac{1}{\left|X^{T}X\right|}\right)$$

The main idea is the same, however we have now three optimal concentrations: zero, maximum and the middle. The optimal solutions can be summarized as follows:

1. D-optimal design puts equal (1/3) number of measurements to these three points
2. To minimize the uncertainty of intercept, one should measure one sample at maximum, one in the middle, then the rest at zero (this also minimizes covariance between uncertainties of the intercept and the linear coefficient).
3. Minimizing the uncertainty of the linear coefficient needs placing half of the samples in the middle and about 1/8 at the maximum concentration.
4. To minimize quadratic coefficient error, half of the samples should still lie in the middle, but the rest divided equally (1/4) to zero and the maximum concentration (this is also very close to A-optimality).
5. A totally different design should be used to minimize the covariances between quadratic coefficient and both other ones: one zero, one in the middle, all other at maximum concentration.

Among the above criteria, D-optimality looks in the most serious way, because it can be perceived as a “compromise” in minimizing all uncertainties with a strong geometrical interpretation: the determinant of covariance matrix ${\left(X^{T}X\right)}^{-1}$ is a measure of a “volume” of the uncertainty cloud in the multivariate space [31,32].

So let us focus on D-optimality criteria, but for a polynomial of any higher order. Surprisingly, the optimal design is then not equal-spaced, but the points are placed more closely to the boundaries of the calibration range.

### 2.5. Fedorov Nonuniform D-Optimal Designs

The analytical solutions can be derived by patient readers inside a computer algebra systems, however the whole problem was solved in general way by Fedorov in 1970s (in the recent edition of his book [33], see Theorem 2.3.3 or take a look at [34]; for other methods of generating D-optimal designs see [35,36]). He discovered that the optimal place of regression points in range <−1, 1> for the fitted equation of *m*-th degree are the roots of $\left(1-x^{2}\right){P^{\prime }}_{m}\left(x\right)$, where $P_{m}\left(x\right)$ is the m-th Legendre polynomial (and the apostrophe operator means the derivative). For the quadratic regression, we have $\left(1-x^{2}\right)x=x-x^{3}$ with roots at −1, 0 and 1 (already known case: the boundaries and the middle). For cubical regression, the polynomial becomes

$$\left(1-x^{2}\right)\left[-15\left(1-x\right)+\frac{15{\left(1-x\right)}^{2}}{2}+6\right]=-\frac{3\left(x-1\right)\left(x+1\right)\left(5x^{2}-1\right)}{2}$$

with nonequally spaced roots x=±1,x=±1/√5, which are equal to 0, $\left(\sqrt{5}\pm 1\right)/\left(2\sqrt{5}\right)$ and 1 after transforming to the range 0–1. Table 1 gives numerical values of D-optimal concentrations computed in analogous way for polynomials up to 9 calibration points on interval 0–1. They were derived by authors with Maxima 5.45.1, using built-in “legendre_p” functions to generate polynomials for solving. They should be transformed to a desired concentration range according to the formula

$$c=c_{\min}+\frac{x}{c_{\max}-c_{\min}}$$

The most important question now is: when should one use higher levels of polynomial for calibration? The answer is: practically never, but the optimal points for higher degrees are in general always better than equally spaced concentrations, even when performing linear or quadratic regression.

### 2.6. Uncertainty of Prediction

Another important idea is to design the calibration experiment to minimize not the uncertainty of the estimators, but the uncertainty of prediction [37,38,39,40], at least at interesting concentrations. The already mentioned hat matrix $H=X{\left(X^{T}X\right)}^{-1}X^{T}$ can be used to calculate the variance of the prediction of *y* for each *x* value. For $x_{i}$, it can be expressed as $\sigma^{2}\left(1+h_{ii}\right)$, where $h_{ii}$ is the i-th diagonal entry and $\sigma^{2}$ is variance of residuals. In matrix form, this can be expressed for all calibration points as $\sigma^{2}[I+X\left(X^{T}X{)}^{-1}X^{T}\right]\;$, where **I** is the identity matrix (ones on diagonal, zeros in the other cells–it is equivalent to add one to each element of the diagonal).

In analogous way to the covariance matrix, we can use three main criteria for optimality in context of the predicted variance [41]:

1. G-optimality minimizes the maximal value of the hat matrix diagonal (thus minimizing the maximal uncertainty)
2. I-optimality minimizes the average uncertainty (expressed for example as the trace of hat matrix)
3. V-optimality minimizes the average uncertainty for the specific range or set of concentrations.
4. Other criteria based on Kiefer approximation theory [42] or Bayesian theory [43] are interested for the enhancement of the reader’s knowledge, but rarely used in the calibration practice.

The prediction variance can be computed in this way for any *x* value, not only these used in calibration. To achieve this goal, we should replace the first and the last elements in hat matrix formula with (analogous to X) matrix containing appropriate *x* values. The number of these values does not matter, so we can form the following formula for computing the predicted variance of one particular *x* in linear calibration [44]:

$$\sigma_{x}^{2}=\sigma^{2}(1+\left[1\;x\right]{\left(X^{T}X\right)}^{-1}\left[1\;x{]}^{T}\right)=\sigma^{2}\left(1+\frac{1}{n}+\frac{{\left(x-\bar{x}\right)}^{2}}{S_{xx}}\right)$$

The most important conclusions from this formula are:

1. The prediction uncertainty is modeled by quadratic (parabolic) dependence, so it is symmetric and unimodal (having exactly one optimal minimum)
2. The minimum occurs when ${\left(x-\bar{x}\right)}^{2}$ is equal to zero. This occurs for the arithmetic mean of all calibration concentrations
3. To minimize prediction error (across the whole calibration range) we should maximize $S_{xx}$. Again, we end up with half of points located at zero, the other at the maximal concentration.

For quadratic calibration, the formula becomes analogous in the matrix way: $\sigma^{2}(1+\left[1\;x\;x^{2}\right]{\left(X^{T}X\right)}^{-1}\left[1\;x\;x^{2}{]}^{T}\right),$ but much more complex when we see its expansion:

$$\sigma_{x}^{2}=\sigma^{2}(1+\frac{x^{4}\Sigma x^{2}-2\Sigma x^{2}\Sigma x^{3}\Sigma x-2\Sigma x^{3}\Sigma x^{2}\Sigma x+2\Sigma x^{4}x\Sigma x-\Sigma x^{2}nx^{4}+2\Sigma x^{3}nx^{3}-\Sigma x^{4}nx^{2}+3{\left(\Sigma x^{2}\right)}^{2}x^{2}-2\Sigma x^{2}\Sigma x^{3}x-\Sigma x^{2}\Sigma x^{4}+{\left(\Sigma x^{3}\right)}^{2}}{\Sigma x^{4}\Sigma x^{2}-2\Sigma x^{2}\Sigma x^{3}\Sigma x-\Sigma x^{2}\Sigma x^{4}n+{\left(\Sigma x^{3}\right)}^{2}n+{\left(\Sigma x^{2}\right)}^{3}})$$

**Figure 1:**
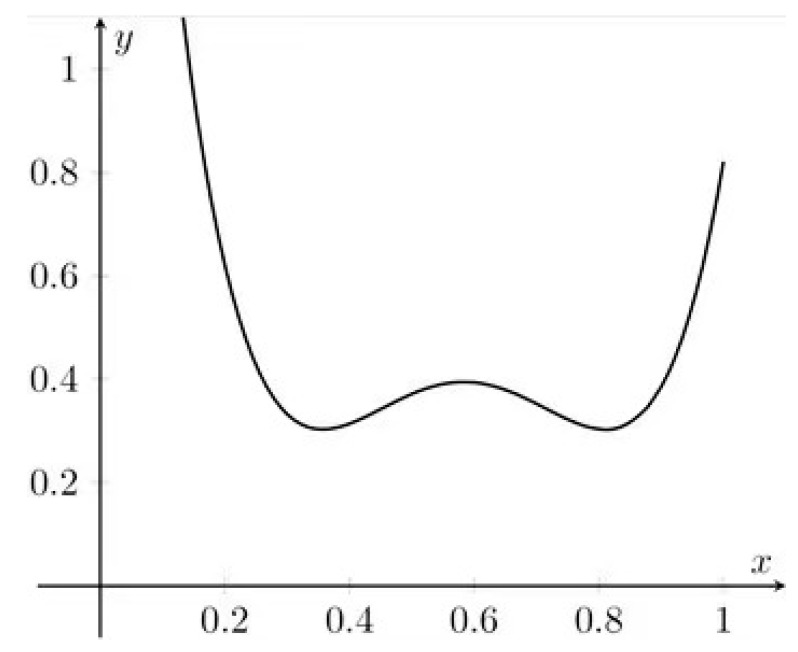
An example of prediction uncertainty pattern for quadratic regression in calibration.

It can be concluded that it is a fourth-degree polynomial, which can have up to three extrema. To see the example, let us consider 6 point uniform calibration curve with concentrations 1/6, 2/6,…, 5/6 and 1. Then:

$$X^{T}X=\left(\begin{array}{ccc} 6 & \frac{7}{2} & \frac{91}{36}\\ \frac{7}{2} & \frac{91}{36} & \frac{49}{24}\\ \frac{91}{36} & \frac{49}{24} & \frac{2275}{1296} \end{array}\right),\;\;\;\;{\left(X^{T}X\right)}^{-1}=\left(\begin{array}{ccc} \frac{16}{5} & -\frac{117}{10} & 9\\ -\frac{117}{10} & \frac{6903}{140} & -\frac{81}{2}\\ 9 & -\frac{81}{2} & \frac{243}{7} \end{array}\right)$$

and we end up with the polynomial (see Figure 1):

$$\left(\begin{array}{ccc} 1 & x & x^{2} \end{array}\right)\left(\begin{array}{ccc} \frac{16}{5} & -\frac{117}{10} & 9\\ -\frac{117}{10} & \frac{6903}{140} & -\frac{81}{2}\\ 9 & -\frac{81}{2} & \frac{243}{7} \end{array}\right)\left(\begin{array}{c} 1\\ x\\ x^{2} \end{array}\right)\;=\;\frac{243}{7}x^{4}-81x^{3}+\frac{9423}{140}x^{2}-\frac{117}{5}x+\frac{16}{5}$$

To obtain its minima, we search for roots of its derivative:

$$\frac{972}{7}x^{3}-243x^{2}+\frac{9423}{70}x-\frac{117}{5}=0$$

The roots are: $\left(\sqrt{185}\pm 35\right)/60$, which corresponds to two minima, as well as 7/12, which corresponds to the maximum between them. The main conclusion is that in a typical design we do not achieve the lowest prediction uncertainty in the middle of the curve. The solution is to redesign the calibration or at least to add several replicates in the interesting region.

## 3. Putting All Together in a Comparison

The reader is now probably significantly confused, as there is no clear answer as to what to do. To compare trends and differences between uncertainty and optimality values, we have chosen 24 samples, as the lowest possible number with rich factorization (giving many combinations of concentration numbers and replicates). We did not consider 2–12 design (two concentrations with twelve replicates of each) as this design can be useful only in linear regression. Therefore, the considered designs are: 3–8, 4–6, 6–4, 8–3 and 12–2. We also did not consider 24–1 combination as its use in practice would be strange.

GNU R 4.1.2 was used as the environment for programming all simulations and for creating graphs. For each design we compared concentrations uniformly spaced between 0 and 1 (including 0 and 1) denoted as U (for example 12–2–U) and nonuniform concentrations satisfying Fedorov theorem, taken from Table 1 and denoted in analogous way with letter L (for example 12–2–L). It should be emphasized, that 3–8–L and 3–8–U are the same design. Table does not contain the concentrations for 12–2 to avoid its widening, they are: 0, 0.0276, 0.0904, 0.1836, 0.3002, 0.4317, 0.5683, 0.6998, 0.8164, 0.9096, 0.9724 and 1. In this way we obtained 10 designs: 5 uniform and 5 nonuniform. They must be shifted in practice to cover range between minimal and maximal concentrations, the formula for this process was given above.

For each of 10 designs we computed: D-optimality for linear and quadratic regression (D-L and D-Q), T-optimality (T-L and T-Q), E-optimality (E-L and E-Q), variance of intercept (S0-L and S0-Q), variance of linear term (S1-L and S1-Q), variance of quadratic term (S2-Q), covariance between the intercept and the linear term (S01-L, S01-Q), covariance between the intercept and the quadratic term (S02-Q) and covariance between the linear and quadratic term (S12-Q). Additionally, we computed maximum prediction variance in the calibration range (G-L and G-Q), average prediction variance in the calibration range (I-L and I-Q) and average prediction variance for range 0.2–0.8 (V-L and V-Q). This gave us 21 measures of optimality for each considered design. Thus, the final optimality matrix had 10 rows and 21 columns.

**Figure 2:**
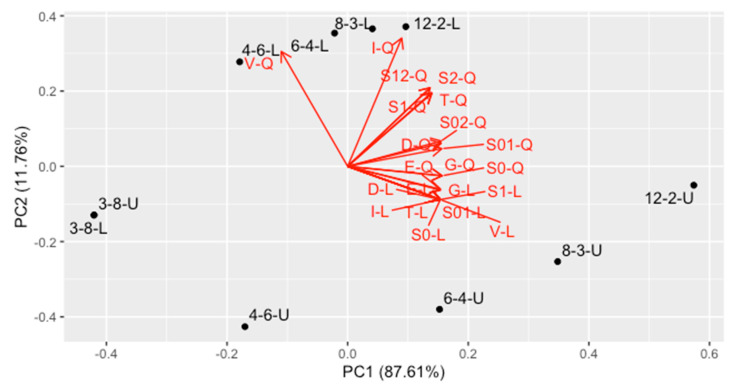
Principal Component Analysis of the various optimality criteria of compared designs. For the abbreviations, see text in Section 3.

The best tool to compare the designs and to visualize trends inside is Principal Component Analysis (PCA). We have computed scaled PCA on this matrix and the results is visualized in Figure 2. As the first two PCs contain 99.4% of variance, almost everything is visualized on two-dimensional PC1-PC2 plot. The following conclusions can be made from this graph:

1. The second PC, containing 11.76% of variance, represents mainly the V-, T- and I-optimality for quadratic regression, together with uncertainty of linear and quadratic coefficient in quadratic regression, as well as covariance between them. They are quite intercorrelated. Designs located at the bottom of the plot are the best ones in this trend.
2. The first PC contains the average optimality for all the other criteria (87.61% variance). This trend contains all criteria for linear model and intercept term for the quadratic one, as well as correlation of the intercept with linear and quadratic term in quadratic regression. Designs located at the left side are the best ones regarding this trend.
3. In general, contrary to the intuitive perception, a design is better when it uses less concentrations and more replicates. The best one is 3–8 design (in the bottom-left corner).
4. The difference between uniform and nonuniform version of each design changes with number of concentrations.
5. For 4 concentrations, the difference is almost vertical, and they are located at the left side of the graph. Therefore, they perform equally well for linear model, but for quadratic model the uniform design is significantly better.
6. For 6 concentrations, the difference for linear model appears, so nonuniform design is visibly better in this case, the difference on vertical axis is analogous.
7. For 8 and 12 concentrations, the difference is more horizontal, and the nonuniform design is much more better than the uniform one.
8. The worst among the considered designs is 12–2-U.

**Figure 3:**
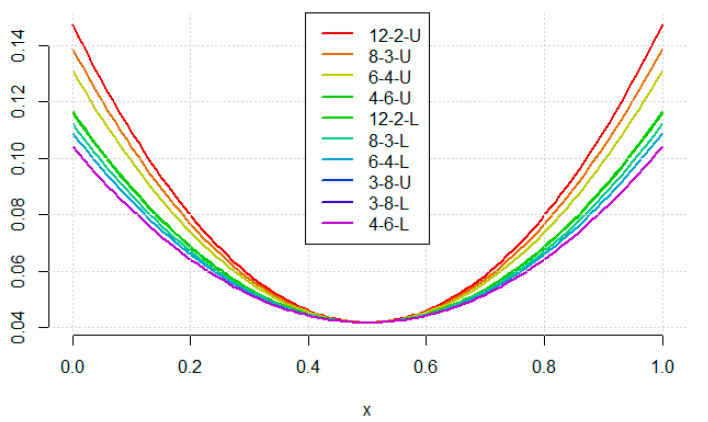
The prediction uncertainty shape for the linear model with the considered designs. For the abbreviations see text in Section 3.

**Figure 4:**
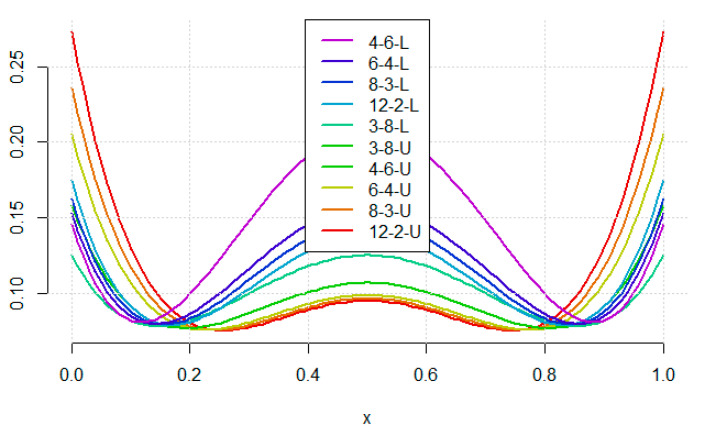
The prediction uncertainty shape for the quadratic model with the considered designs. For the abbreviations see text in Section 3.

The shapes of predicted variances along the calibration range are presented in Figure 3 (for linear model) and Figure 4 (for quadratic model). For the linear curve, it can be seen that the minimal prediction uncertainty is always obtained in the middle of the graph (the arithmetic mean of the concentrations) and its value does not differ among designs. The curve is unimodal, so all three prediction criteria rank the designs in the same way (that is why the loading arrows for these criteria in Figure 2 are pointing in the same direction).

For the quadratic curve, the situation is more complex. The lowest prediction uncertainty is achieved around concentrations 0.2 and 0.8 and it increases in the middle of the calibration range. Lowering uncertainty in the middle causes substantial increase in the boundaries of calibration range. Therefore, minimizing the average or maximal uncertainty along the whole prediction range is not equivalent to minimizing uncertainty in the middle. The V-optimality (for the range 0.2–0.8) is the most reasonable criterion here, as the prediction is done mainly in this range. The rankings of designs are consistent with these done with PCA.

## 4. Conclusions

Concluding, if an analyst can perform 24 calibration samples, the best possible design is 3–8, however it does not allow to detect more complex than quadratic nonlinearity [45]. Therefore, it can be used only in routine calibration, when the shape of the calibration dependence was detected in the earlier calibration routines. The best compromise could be considered as 6–4-L. If more concentrations are required for some reason, one should consider 8–3-L or 12–2-L. Uniform designs for larger number of concentrations perform visibly worse, so one should not consider 6–4-U, 8–3-U and 12–2-U.

## Figures and Tables

**Table 1:** D-optimal nonuniform designs for standardized interval 0–1.

Concentrations									
2	0	1							
3	0	0.5000	1						
4	0	0.2764	0.7236	1					
5	0	0.1727	0.5000	0.8273	1				
6	0	0.1175	0.3574	0.6426	0.8825	1			
7	0	0.0849	0.2656	0.5000	0.7344	0.9151	1		
8	0	0.0641	0.2041	0.3954	0.6046	0.7959	0.9359	1	
9	0	0.0501	0.1614	0.3184	0.5000	0.6816	0.8386	0.9499	1
